# Trafficking and localization of *KNOTTED1* related mRNAs in shoot meristems

**DOI:** 10.1080/19420889.2022.2095125

**Published:** 2022-07-06

**Authors:** Munenori Kitagawa, Xiaosa Xu, David Jackson

**Affiliations:** Cold Spring Harbor Laboratory, Cold Spring Harbor, NY, USA

**Keywords:** KNOTTED1, SHOOT MERISTEMLESS, mRNA trafficking, plasmodesmata, meristem, L1 layer, maize, arabidopsis

## Abstract

Multicellular organisms use transcripts and proteins as signaling molecules for cell-to-cell communication. Maize KNOTTED1 (KN1) was the first homeodomain transcription factor identified in plants, and functions in maintaining shoot stem cells. KN1 acts non-cell autonomously, and both its messenger RNA (mRNA) and protein traffic between cells through intercellular nanochannels called plasmodesmata. KN1 protein and mRNA trafficking are regulated by a chaperonin subunit and a catalytic subunit of the RNA exosome, respectively. These studies suggest that the function of KN1 in stem cell regulation requires the cell-to-cell transport of both its protein and mRNA. However, *in situ* hybridization experiments published 25 years ago suggested that *KN1* mRNA was missing from the epidermal (L1) layer of shoot meristems, suggesting that only the KN1 protein could traffic. Here, we show evidence that *KN1* mRNA is present at a low level in L1 cells of maize meristems, supporting an idea that both KN1 protein and mRNA traffic to the L1 layer. We also summarize mRNA expression patterns of KN1 homologs in diverse angiosperm species, and discuss KN1 trafficking mechanisms.

Cell-to-cell communication is essential for determining cell fates, and is the basis for multicellular development. For example, stem cells divide to self-renew and produce cells destined to differentiate, and many forms of cell-to-cell communication regulate their identity and proliferation [1,2]. Plants use multiple types of cell-to-cell signaling, including secreted ligands and receptors, as well as direct transfer of molecules through plasmodesmata, membrane-lined nanochannels that penetrate the cell wall [3–5]. Plasmodesmal signaling is critical for maintaining plant stem cell niches, or meristems [6–8]. Several transcription factors, including homeodomain factors, act as non-cell-autonomous signals by trafficking through plasmodesmata [9].

Maize KNOTTED1 (KN1) was the first homeodomain transcription factor identified in plants, and the first transcription factor found to traffic via plasmodesmata [10,11]. *KN1* homologs, so-called class I *KN1*-like homeobox (*KNOX* I) genes, are conserved in all taxa in the plant kingdom [12,13]. The primary function of *KNOX* I genes is to maintain the pool of stem cells in shoot meristems, as shown by the loss of meristems in maize *kn1* mutants [14–16]. This function, as well as cell-to-cell mobility, is conserved widely, for example, in the *KN1* homolog *SHOOT MERISTEMLESS* (*STM*) in *Arabidopsis* [17–19]. While transcription factor protein trafficking is broadly documented, the function of class I *KNOX* genes requires trafficking of both their protein and mRNA [7,8,19]. Regulators of class I KNOX protein and mRNA trafficking, such as chaperonins and an RNA exosome subunit, respectively, and additional mobile transcription factors, such as WUSCHEL and SHORT-ROOT, have been identified [7,8,20–22].

In addition to short-range cell-to-cell trafficking, proteins and mRNAs are also selectively transported systemically between plant organs via the phloem. Regulatory factors and protein/RNA motifs and modifications important for this long-range transport have also been identified [23,24]. Thus, cell-to-cell signaling using proteins and mRNAs is a rapidly developing field, and although significant progress has been made in understanding its mechanisms, there are still many open questions.

Previous studies suggested that KN1 protein and mRNA interact as they traffic between cells, perhaps by forming a ribonucleoprotein (RNP) complex [11,25,26]. If KN1 and STM traffic as RNPs, they may need to streamline their shape to pass through the tiny plasmodesmata pores. Chaperones and RNA helicases may be involved in this process [27,28]. This process may also involve RNA-binding proteins that function as carriers, and their receptors, as well as actin and myosin that can alter plasmodesmal pore size [27,29,30]. In our recent study, we found that a catalytic subunit of the RNA exosome, *Arabidopsis* Ribosomal RNA-Processing Protein 44A (AtRRP44A), controls *KN1* and *STM* mRNA trafficking between cells [8]. AtRRP44A is predominantly nuclear, but when levels in the cytoplasm are enhanced by the addition of a nuclear export sequence, it has a capacity to localize to plasmodesmata. These findings suggest that AtRRP44A is involved in the plasmodesmata targeting of class I KNOX RNPs, the conversion of RNPs to a mobile form, or the trafficking through plasmodesmata. In support of these ideas, we found that *KN1* mRNAs localize to cytoplasmic puncta that move dynamically around the cytoplasm, and transiently interact with plasmodesmata [8]. This interaction could allow *KN1* mRNA to traffic through plasmodesmata to neighboring cells. However, how *KN1* mRNA is targeted to plasmodesmata is unknown. The mRNA of another mobile factor, FLOWERING LOCUS T, is tethered to endosomes and recruited to plasmodesmata via microtubules and actin [31]. Since STM is also associated with endosomes and microtubule-associated proteins [20,21], it may be targeted to plasmodesmata by a similar mechanism.

The trafficking of KN1 and STM proteins and RNAs has been studied mostly in *Arabidopsis* and tobacco leaves, but how they traffic in the shoot meristem, where they function, is less well understood. However, mutants that reduce KN1/STM protein or mRNA trafficking in the leaf, such as chaperonin or RNA exosome subunits mutants, significantly affected meristem development [7,8,19], suggesting their trafficking in the meristem is important for normal development. Angiosperm shoot meristems have a layered structure, where an outer epidermal L1 layer covers inner layers. Despite multiple reports of *KN1* and *STM* mRNA trafficking, the original report of KN1 trafficking presented contradictory results, as *KN1* mRNA was detected in the inner meristem layers but absent from the L1, whereas KN1 protein was detected throughout all meristem layers [32,33]. This difference in localization led to the prediction, and later demonstration, that KN1 protein can traffic from the inner meristem layers to the L1 [11]. However, the original report and several others suggested that KN1 traffics with its mRNA as an RNP [8,25]. Homeodomain proteins are known for their DNA binding activity, but their specific mRNA binding has also been demonstrated in flies [34,35]. However, if *KN1* mRNA can traffic, and KN1 protein and mRNA can form an RNP, it is puzzling that *KN1* mRNA is not detected in the L1 layer of the maize shoot meristem. One possible explanation is that KN1 RNPs traffic between cells in the inner meristem layers, but only KN1 protein traffics to L1 [36], however, this seems unlikely. Another possibility is that *KN1* mRNA does traffic to the L1, but its levels there are too low to be detected by *in situ* hybridization. Even a few *KN1* mRNA molecules in the L1 could be amplified by multiple rounds of translation to produce abundant protein levels [37,38]. Indeed, we present evidence here that this is likely to be the case.

Recently, single-cell mRNA sequencing (scRNA-seq) has provided unprecedented resolution in plant expression studies [39–41]. In a scRNA-seq experiment of developing maize ears, we found multiple distinct cellular clusters representing known cell types and domains, and indeed we found *KN1* transcripts in meristem L1 cells [42,43] (Figure 1(a)). However, these transcripts could be background noise or sporadic expressions captured in the scRNA-seq experiments. A recent laser microdissection (LCM) RNA-seq experiment also detected *KN1* transcripts in L1 cells of the shoot meristem. The *KN1* mRNA levels in the L1 were about one tenth of those in the L2, but much higher than in leaf primordia, where *STM* expression is repressed [44]. To support these findings, we performed *KN1 in situ* hybridization [32] using a longer detection period. Indeed, we detected weak *KN1* mRNA *in situ* signal in L1 cells (Figure 1(b)). While we cannot rule out the possibility that this signal is from diffusion of the alkaline phosphatase reaction product, the combined evidence of scRNA-seq, LCM and mRNA *in situ* hybridization supports the idea that a small amount of *KN1* mRNA traffics from the inner meristem layers to the L1.

**Figure 1.**
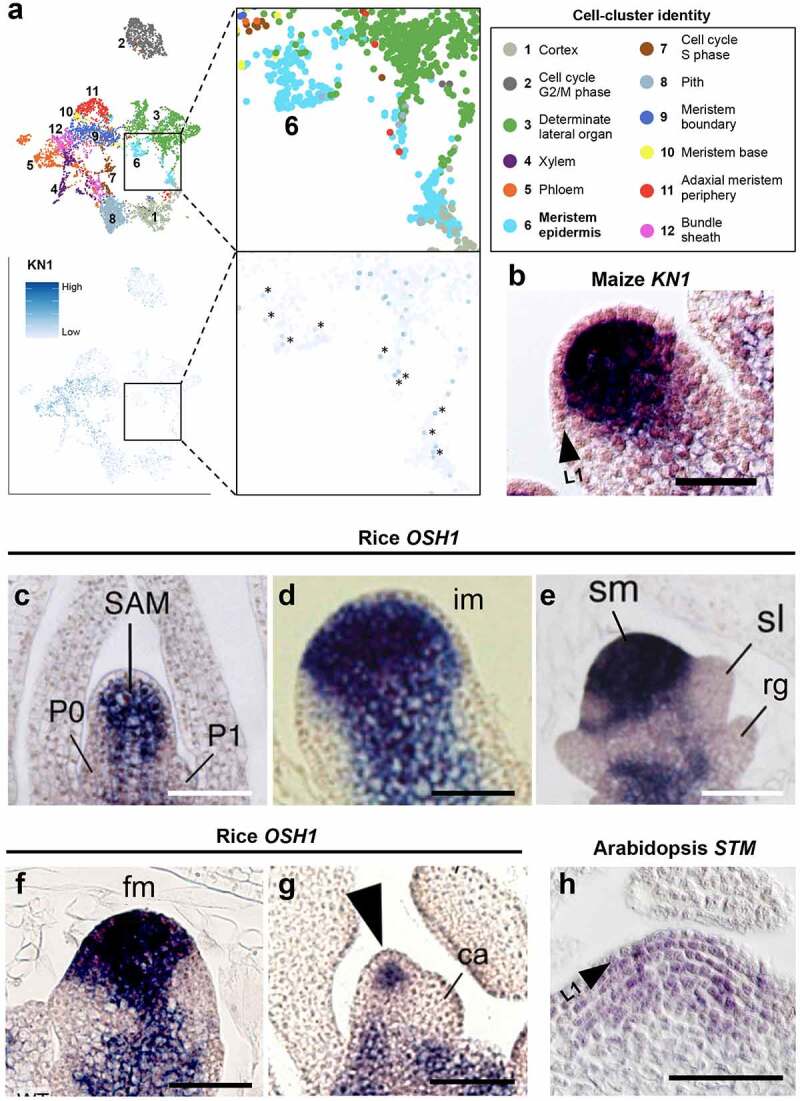
*KN1* mRNAs are detected at low levels in L1 (epidermal) cells of maize meristems. (a) Single-cell RNA sequencing [42] indicates that *KN1* transcripts are abundant in meristem (clusters 9, 10, and 11), vasculature (clusters 4, 5, and 12), and ground tissue (clusters 1 and 8), but also present at low levels in meristem L1 cells (cluster 6, asterisks). (b) Over-exposure of a *KN1* mRNA *in situ* hybridization shows a weak signal in the L1 (pink) and a strong signal in the inner meristem layers (dark blue) in a maize ear spikelet pair meristem. (c-g) Rice *OSH1* mRNA is absent from the L1 layer of the vegetative shoot apical meristem (SAM) (c) but observed in some L1 cells in the inflorescence meristem (im) (d), and is throughout the L1 in the spikelet meristem (sm) (e) and floret meristem (fm) (f), then is again restricted to the inner layers in the later stage fm (g). P0 and P1, plastochron 0 and 1; rg, rudimentary glume; sl, sterile lemma; ca, carpel. (h) mRNA *in situ* hybridization showing *STM* mRNA in the entire vegetative shoot meristem including L1 layer in *Arabidopsis*. The data used for panel A is from [42]. Panel C, D, E, F-G, and H used images from [48,54–56] and [8] with permission, respectively. Scale bars = 50 µm.

It is also interesting to compare expression patterns of KN1 and STM homologs in diverse angiosperm species. Expression varies significantly between species and meristem stages, suggesting interesting hypotheses about the regulation of trafficking of KN1/STM-related transcripts. In maize, *KN1* mRNA appears to be restricted to the inner meristem layers in both vegetative and inflorescence stages, and is mostly undetectable in the L1 layer [32] except as described above. Similar patterns are seen in other species, including in brachypodium spikelet and floral meristems and wheat vegetative meristems [45,46]. In some species, however, expression is clearly observed in the L1 layer at particular stages of development. For example, mRNA of the rice *KN1* ortholog *ORYZA SATIVA HOMEOBOX1* (*OSH1*) localizes to the inner meristem layers of vegetative and inflorescence meristems, but is also observed in the L1 meristem layer in spikelet and early stage flower meristems. However, expression is once again restricted to the inner meristem layers in the late stage flower meristems [47,48] (Figure 1(c-g)). In tomato and tobacco, *KN1* ortholog mRNAs are also restricted to the inner cell layers in vegetative meristems, but are clearly detected in the L1 layer at the reproductive stages [49–51]. Thus, localization of *KN1* homolog transcripts is often excluded from the L1 layer in vegetative stages, but found in the L1 layer in later stages. A different situation is observed for *Arabidopsis* STM, where its mRNA is not detected in the L1 in early embryo stages, but is detected there in later embryo and seedling and reproductive stages [17] (Figure 1(h)). What causes these changes in mRNA localization between species and meristem stages? One possibility is that *KNOX* I gene transcription switches between layers depending on the species and/ or developmental stage. However, another possibility is that the mobility of *KNOX* I mRNA between cell layers is differentially regulated. In support of this idea, the permeability and number of plasmodesmata change dynamically during meristem transitions [52], and this might affect selective transport of specific transcripts. A better understanding of these processes could enable manipulation of KNOX expression and localization to fine-tune meristem activity, and improve plant growth and crop yields.
